# Nasopharyngeal Actinomycosis

**DOI:** 10.1155/2011/367364

**Published:** 2011-10-24

**Authors:** Lamia Ouertatani, Yassine Jeblaoui, Salima Kharrat, Samia Sahtout, Ghazi Besbes

**Affiliations:** Service d'ORL et de Chirurgie Maxillo-Faciale, Hopital la Rabta, Tunis 1007, Tunisia

## Abstract

Nasopharyngeal actinomycosis is a rarely encountered bacterial infection which usually occurs after nasal trauma or surgery. In some clinical cases, nasopharyngeal actinomycosis has appeared in patients without prior trauma, making diagnosis difficult. Here we present three such cases successfully treated with appropriate dosages of penicillin. One 16-year-old boy with no previous medical antecedents showed an important thickening of the posterior wall of the nasopharynx. A similar nasopharyngeal thickening was found in a 42-year-old woman exhibiting poor dental hygiene. In another 42-year-old woman, nasopharyngeal inflammation was accompanied by multiple right lymphoadenopathies. Like the first two patients, the woman had no prior trauma but did exhibit poor dental hygiene and teeth rottenness. In all three patients, actinomycosis diagnosis was confirmed by anaerobic microbial culturing of the biopsy specimen. Although diagnosis is delayed in patients with no prior trauma, treatment with antibiotics has greatly improved the prognosis for all forms of actinomycosis, and neither death nor deformity is common.

## 1. Introduction

Actinomycosis is a subacute-to-chronic bacterial infection caused by filamentous, gram-positive, anaerobic-to-microaerophilic bacteria that are not acid fast. It is characterized by contiguous spread, suppurative and granulomatous inflammatory reaction, and formation of multiple abscesses and sinus tracts that discharge sulphur granules. The most common clinical forms of actinomycosis are cervicofacial (i.e., lumpy jaw), thoracic, and abdominal. In women, pelvic actinomycosis is common. 

Nasopharyngeal actinomycosis is a rare clinical disease. It can occur after nasal trauma or surgical manipulation [1]. It is also reported to occur without prior trauma, making diagnosis difficult [2].

## 2. Case  1

**Figure 1:**
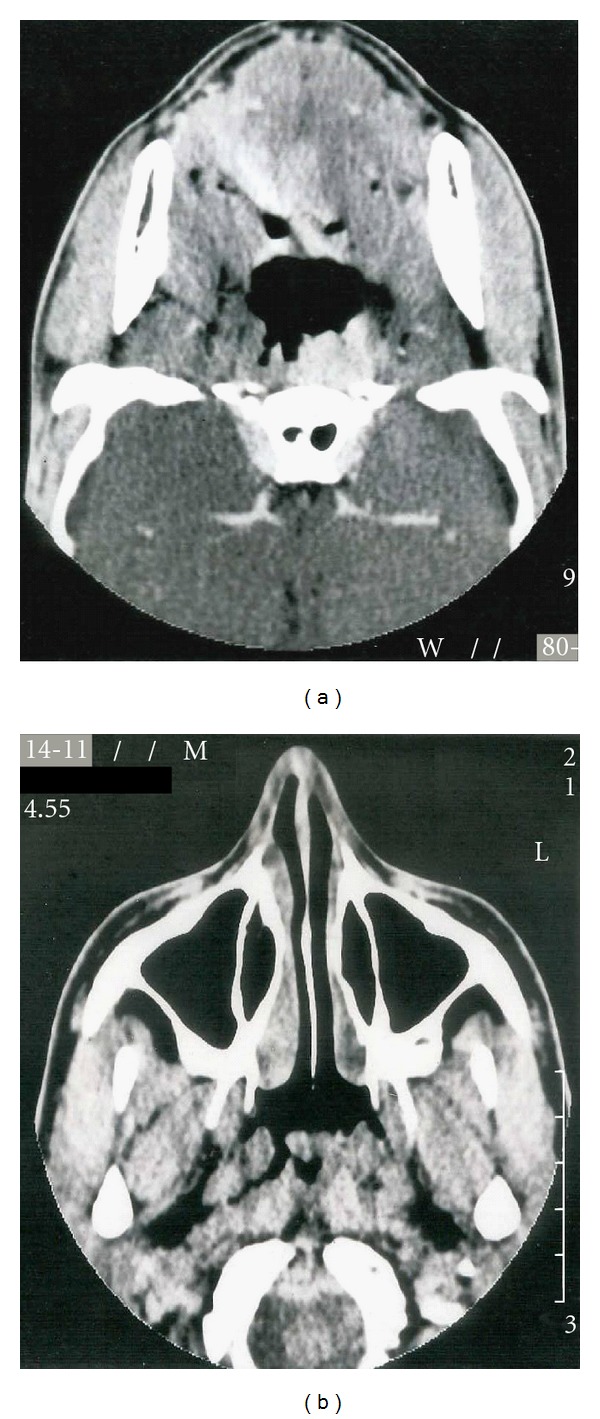
Facial CT: thickening of the left nasopharynx.

A 16-year-old man was referred with a 6-month history of worsening hyponasality and a seropurulent otorrhoea. He had an unremarkable past medical history. He denied weight loss, dyspnea, epistaxis, or fever. The clinical evaluation revealed significant thickening of the posterior wall of the nasopharynx. There was no lymphadenopathy present. A CT scan showed thickening of the left nasopharynx (Figure 1). A biopsy was performed. The histopathology revealed chronic inflammation and the presence of actinomyces. Gram stains of the tissue revealed gram+ with filamentous organisms oriented radially around sulphur granules.

The patient was diagnosed with actinomycosis and successfully treated with a prolonged course of penicillin (45 days). He subsequently improved and recovered after 34 months. The evaluation of the treatment outcome was made by nasopharyngoscopy. No further biopsy or CT scan was made. 

## 3. Case  2

A 42-year-old woman without past medical history was referred for a right neck mass associated to leanness without rhinologic or otologic problems.

Physical examination revealed a right subdigastric 3 cm × 5 cm nontender and firm neck node. The remainder of the head and neck examination was unremarkable except for the finding of poor dental hygiene. The blood cell count was normal. The nasopharyngoscopy showed a nonulcerated mass of the right nasopharynx. Ultrasound imaging of the neck revealed right cervical lymphadenopathy with the largest node measuring 4 cm. The CT scan showed a thickening of the nasopharynx wall with multiple right lymphadenopathy. 

**Figure 2:**
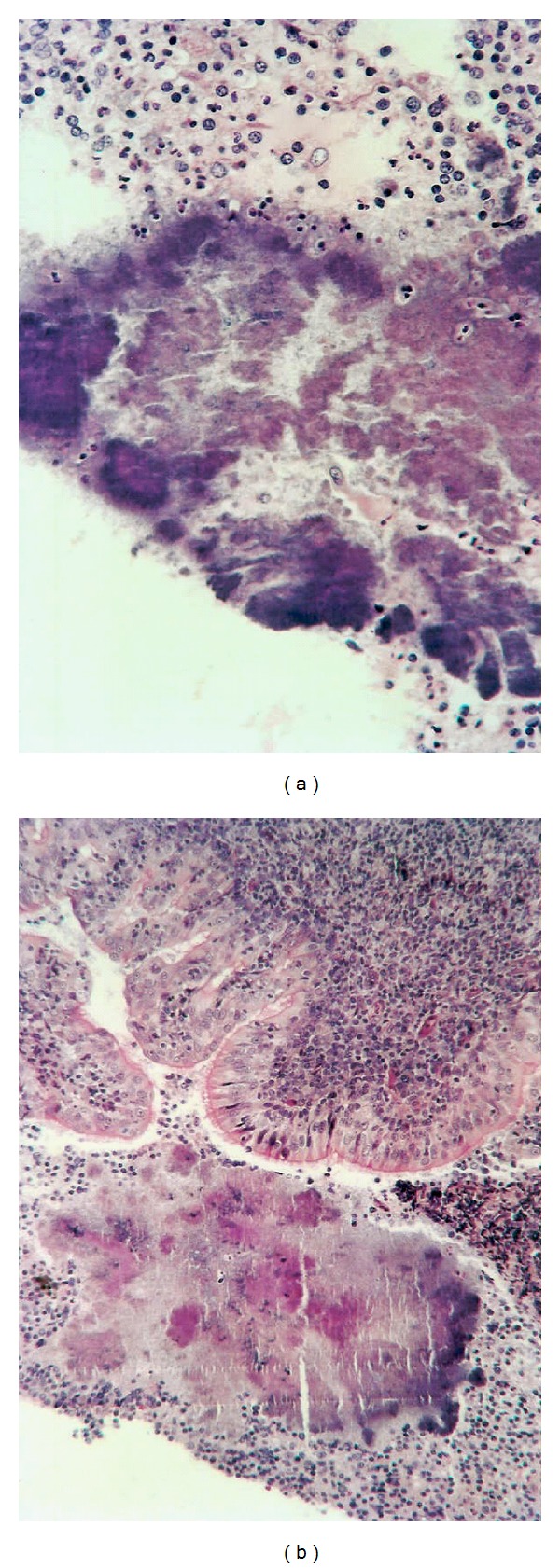
Chronic inflammation and presence of actinomyces.

The histopathology revealed the presence of actinomyces (Figure 2). She was diagnosed with actinomycosis and treated with high-dose penicillin, intravenously followed by oral penicillin during 2 months. She finally recovered after 10 months.

## 4. Case  3

A 42-year-old woman was referred for headache and epistaxis without nasal obstruction. Physical exam showed poor dental hygiene. The nasopharyngoscopy revealed a thickening of the right nasopharynx. The biopsy showed the presence of actinomyces. She was diagnosed with actinomycosis and treated with penicillin until recovery. (20 M/d during 45 days).

## 5. Discussion

Nasopharyngeal actinomycosis is very rare. Only a few cases have previously been published. Actinomyces was first clinically described in 1857. They are prominent among the normal flora of the oral cavity and less prominent in the lower gastrointestinal tract and female genital tract [3]. They are isolated in 29% of sample of saliva in healthy people [4] and most commonly affect the cervicofacial region in 40 to 70% of cases [5].

Only a few cases of actinomycosis of the nasopharynx are reported in the literature, most resulting from mucosal trauma [6]. In our cases, no traumatic event could be elucidated.

Improved dental hygiene and widespread use of antibiotics for various infections probably have contributed to the declining incidence of this disease. As these microorganisms are not virulent, they require a break in the integrity of the mucous membranes and the presence of devitalized tissue to invade deeper body structures and cause human illness [6]. 

Furthermore, actinomycosis is generally a polymicrobial infection, with isolated numbering as many as 5–10 bacterial species [4]. Establishment of human infection may require the presence of such companion bacteria, which participate in the production of infection by elaborating a toxin or enzyme or by inhibiting host defences. These companion bacteria appear to act as copathogens that enhance the relatively low invasive power of actinomycetes. Specifically, they are responsible for the early manifestations of the infection and for treatment failures.* Actinomyces Israelii *is the most common human pathogen. It is a gram-positive, filamentous, slow-growing anaerobic bacterium. The characteristic sulphur granule consists of a small colony of intertwined, branching actinomyces filaments solidified with elements of tissue exudates, grossly resembling a grain of sulphur [4]. 

Once infection is established, the host mounts an intense inflammatory (i.e., suppurative, granulomatous) response, and fibrosis develops subsequently. Patients present with nodular lesion(s) which gradually increase in size and number (i.e., multiple abscesses). Sulphur granules may be seen in the exudate.

Nodules may be tender in the initial stages, but typically they are nontender and woody hard in the later stages.

Trismus is present if the muscles of mastication are involved. Fever is variably present. Infection typically spreads contiguously, frequently ignoring tissue planes and invading surrounding tissues or organs. Ultimately, the infection produces draining sinus tracts. Hematogenous dissemination to distant organs may occur in any stage of the infection, whereas lymphatic dissemination is unusual. 

There are two main forms of actinomycosis. The more common presentation is a chronic, slowly progressive, indolent infection that causes indurated infiltration and multiple abscesses and fistula [7]. In some patients, actinomycosis is an acute, rapidly progressive infection associated with pain, fever, soreness, and swelling. Fistula formation, commonly seen in cervicofacial actinomycosis, has not been observed in nasopharyngeal actinomycosis. The delay of diagnosis is relatively long varying from 10 days to one year [2].

Actinomycosis can affect people of all ages, but the majority of cases are reported in young to middle-aged adults (aged 20–50 y) [1]. Two of our patients were aged 42 years, the third was 16 years old. 

Young adult males are commonly affected, attributed to a preponderance of males being involved in accidents and fights causing maxillofacial trauma and thus inoculation of bacteria. The reported male-to-female ratio is 3/1 [8]. Our patients' sex ratio was 1/2. No racial predilection exists [4]. Anaemia and mild leukocytosis are common. Erythrocyte sedimentation rate (ESR) is often elevated.

Diagnosis of actinomyces is done by culturing. However, only 50% of cultures from cases highly suspicious for actinomycosis grow the elusive organism. This is thought to be because of the necessity of strict anaerobic culturing conditions. Recently, monoclonal antibody staining with fluorescent conjugated monoclonal antibodies has become available to detect actinomyces.

Imaging studies can be helpful to determine exact location and extent of involvement as well as determining bony destruction, although there is no uniform characteristic that can absolutely determine diagnosis. CT scans usually reveal an infiltrative mass with focal areas of decreased attenuation that enhance with contrast. This infiltrative mass has a tendency to invade surrounding tissues; surrounding lymphadenopathy is uncommon. 

Treatment consists of surgical debridement and extended antibiotic therapy. High-dose penicillin is the antibiotic of choice [9]. The duration of treatment may be variable among different centres but a treatment of one to three months is recommended.

When actinomycosis is diagnosed early and treated with appropriate antibiotic therapy, the prognosis is excellent.

The more advanced and complicated actinomycotic forms require aggressive antibiotic and surgical therapy for optimal outcome; however, deaths can occur despite such therapy [10].

## 6. Conclusion

Actinomycosis of the nasopharynx is a rare entity. Diagnosis is difficult and delayed and is made after identifying the bacteria in biopsy specimens. Treatment consists of prolonged antibiotic therapy. The availability of antibiotics has greatly improved the prognosis for all forms of actinomycosis. At present, cure rates are high and neither deformity nor death is common.

## References

[1] Nagler R, Peled M, Laufer D (1997). Cervicofacial actinomycosis: a diagnostic challenge. *Oral Surgery, Oral Medicine, Oral Pathology, Oral Radiology, and Endodontics*.

[2] Chiang CW, Chang YL, Lou PJ (2000). Actinomycosis imitating nasopharyngeal carcinoma. *Annals of Otology, Rhinology and Laryngology*.

[3] Smego RA, Foglia G (1998). Actinomycosis. *Clinical Infectious Diseases*.

[4] Bennhoff DF (1984). Actinomycosis: diagnostic and therapeutic considerations and a review of 32 cases. *Laryngoscope*.

[5] Miller M, Haddad AJ (1998). Cervicofacial actinomycosis. *Oral Surgery, Oral Medicine, Oral Pathology, Oral Radiology, and Endodontics*.

[6] Osborne JE, Blair RL, Christmas HE, McKenzie H (1988). Actinomycosis of the nasopharynx: a complication of nasal surgery. *Journal of Laryngology and Otology*.

[7] Burns BV, Al-Ayoubi A, Ray J, Schofield JB, Shotton JC (1997). Actinomycosis of the posterior triangle: a case report and review of the literature. *Journal of Laryngology and Otology*.

[8] Houman MH, Ben Ghorbel I, Rammah Ben Achour N (2001). Vertebral actinomycosis with spinal cord compression. A case report. *Revue de Medecine Interne*.

[9] Daamen N, Johnson JT (2004). Nasopharyngeal actinomycosis: a rare cause of nasal airway obstruction. *Laryngoscope*.

[10] Scott A (1997). Actinomycosis presenting as a nasopharyngeal tumour: a case report. *Journal of Laryngology and Otology*.

